# The evaluation of contralateral foot circulation after unilateral revascularization procedures using indocyanine green angiography

**DOI:** 10.1038/s41598-017-16527-7

**Published:** 2017-11-23

**Authors:** Masahiro Nakamura, Kimihiro Igari, Takahiro Toyofuku, Toshifumi Kudo, Yoshinori Inoue, Hiroyuki Uetake

**Affiliations:** Department of Surgical Specialties, Graduate School of Medical and Dental Sciences, Tokyo Medical and Dental University, 1-5-45, Yushima, Bunkyo-ku, Tokyo, 113-8519 Japan

## Abstract

The aim of the present study is to assess the effects of unilateral revascularization on the contralateral foot circulation using indocyanine green (ICG). From January 2016 to April 2016, a total of twenty-one patients were included in this study. The patients underwent elective unilateral revascularization at our institution and we evaluated the feet circulation by indocyanine green angiography (ICGA) tests preoperatively and postoperatively. The ICGA parameters included the magnitude of intensity from the onset of ICG to the maximum intensity (I_max_), the time from the onset of ICG to the maximum intensity (T_max_), and the time required to reach the half maximum intensity from the onset of ICG (T_1/2_). There were significant differences in the treated limb T_max_ (*P* = 0.016) and T_1/2_ (*P* = 0.013) values and in the contralateral limb T_max_ (*P* = 0.013), and T_1/2_ (*P* < 0.001) values on the perioperative ICGA tests. These results reflect the increase in skin perfusion in the treated limb and the decrease in skin perfusion in the contralateral limb. Unilateral revascularization decreases contralateral foot circulation. The preoperative contralateral lesion should be evaluated when revascularization is performed.

## Introduction

Lower extremity revascularization procedures are performed in patients with peripheral arterial disease (PAD) with the aim to treat ischemia. Although contralateral symptoms often develop after unilateral revascularization, this may be due to the progression of the atherosclerotic lesion or latent symptoms that are uncovered because the treatment allows the patients to walk long enough to recognize the contralateral symptoms^1^. On the other hand, unilateral revascularization for critical limb ischemia (CLI) has been previously reported to induce decreased skin perfusion in the contralateral foot^2^. However, the mechanism of underlying this phenomenon is not clear and has never been fully investigated. A previous study showed that unilateral CLI and a poor contralateral ankle brachial pressure index (ABI) value at the time of the initial revascularization are associated with a high risk of contralateral CLI after revascularization^1^.

Macrovascular circulation assessments including the ABI, toe-brachial pressure index (TBI), and toe pressure (TP) can evaluate the effects of revascularization. Indocyanine green (ICG) has been used to estimate liver function^3^ and the viability of skin flaps^4,5^. Recently, ICG has been used to assess skin perfusion in the lower extremities of patients with PAD^6–8^. ICG angiography (ICGA) can evaluate the microcirculation^9–12^, and the hemodynamic changes in the ipsilateral leg that occur due to unilateral revascularization might affect the microvascular circulation of the contralateral leg.

The aim of the present study was to assess the effects of unilateral revascularization on the contralateral lower extremity and to investigate the factors associated with this phenomenon by ICGA.

## Material and Methods

### Patients

The present study was approved by the local ethics committee at Tokyo Medical and Dental University (No. 2332), and written informed consent obtained from each of the subjects. Furthermore, we confirmed that all of the methods were performed in accordance with the relevant ethical guidelines and regulations. From January 2016 to April 2016, 48 patients underwent elective revascularization for PAD at Tokyo Medical and Dental University. The inclusion criteria were (1) patients who had undergone ICGA tests in both the preoperative and postoperative periods; (2) patients who had undergone unilateral revascularization procedures. However, any patients who underwent revascularization procedures that could lead to contralateral hemodynamic changes, such as endovascular treatment (EVT) with access from contralateral side or femorofemoral bypass procedure, were excluded. A total of 21 patients were retrospectively included in the present study. The risk factors we evaluated included hypertension, dyslipidemia, coronary artery disease, cerebrovascular disease, diabetes, chronic renal failure with hemodialysis. Hypertension was defined by the presence of any of the following conditions: systolic blood pressure >140 mmHg, diastolic blood pressure >80 mmHg or treatment with antihypertensive drugs. Dyslipidemia was defined by any of the following conditions: a serum LDL cholesterol >140 mg/dl, HDL cholesterol <40 mg/dl, triglycerides >150 mg/dl or treatment with antilipidemic drugs. Coronary artery disease was defined as a history of angina pectoris, myocardial infarction, or both and was documented by coronary angiography or a history of revascularization of any of the coronary arteries. Cerebrovascular disease was defined as a history of stroke, transient ischemic attack, carotid artery revascularization or cerebral hemorrhage. Diabetes was defined as insulin dependence or treatment with oral antihyperglycemic drugs or diet.

### The assessment of revascularization procedures and hemodynamic change

All patients underwent computed tomography angiography or duplex ultrasound sonography to evaluate the arterial lesions on both legs. The perioperative ABI, TBI and TP values of both legs were measured using a VaSera VS-1500E^TM^ device (FUKUDA DENSHI, Tokyo, Japan) to assess the hemodynamic status. Clinical success was defined by a >0.1 increase in the ABI value or at least a single Rutherford category^13^ improvement of the treated leg after undergoing revascularization procedures.

### ICGA testing

The ICGA tests performed in this study has previously been described in detail^14^. Briefly, the patient was placed in the supine position and a 0.1 mg/kg dose of ICG (Diagnogreen^TM^; Daiichi-Sankyo Pharmaceutical, Tokyo, Japan) was injected intravenously through an upper extremity venous line. Immediately after the injection of ICG, fluorescence imaging was initiated. The images were recorded for five minutes using an infrared camera system (Photodynamic Eye^TM^, Hamamatsu Photonics K.K., Hamamatsu, Japan) that was positioned 20 cm above the dorsal surface of the feet. This study differed from the previous study in that both feet were assessed simultaneously. Preoperative ICGA was performed one or two days before the operation and postoperative ICGA was performed on postoperative day three.

### The ICGA image analysis

**Figure 1:**
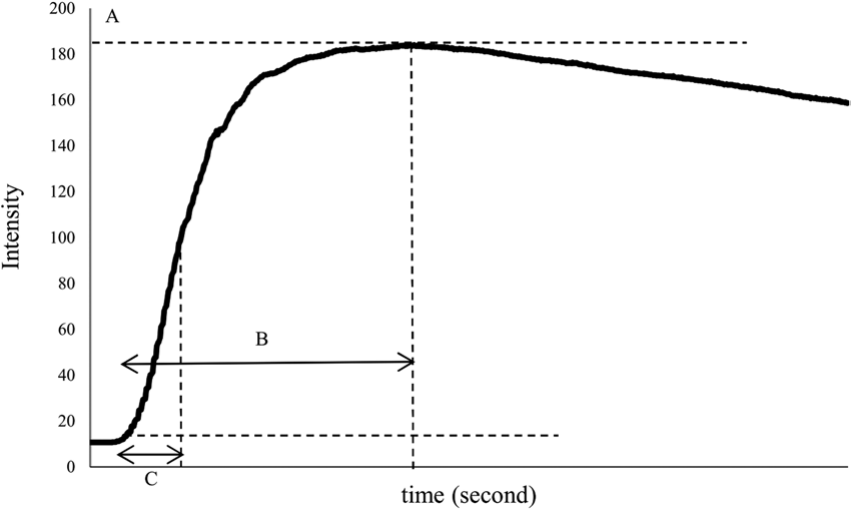
Indocyanine green angiograpy test parameters were defined graphically. (**A**) I_max_; (**B**) T_max_; (**C**) T_1/2_. *Note*. I_max_ indicated the magnitude of intensity from the onset of ICG to maximum intensity; T_max_ indicated the time from the onset of ICG to the maximum intensity, and the T_1/2_ indicated the time required to reach the half-maximum intensity from the onset of ICG.

A region of interest (ROI) was set on both feet to compare the perioperative skin perfusion. The ROI was the whole dorsum of the foot from the transverse tarsal joint to the distal part of the metatarsal bones. Three parameters were measured in the ROI to evaluate skin perfusion: the magnitude of intensity from the onset of ICG to the maximum intensity (I_max_), the time from the onset of ICG to the maximum intensity (T_max_), the time required to reach the half-maximum intensity from the onset of ICG (T_1/2_) (Fig. 1). To investigate the factors associated with the change in skin perfusion in the contralateral foot, the rate of change (RC) was calculated as the ratio of the postoperative parameters to the preoperative parameters of the bilateral feet.

### Statistical analysis

Continuous variables are expressed as the median and inter-quartile range (IQR). Statistical significance was assessed using the Wilcoxon signed-rank test or Spearman’s rank correlation coefficients to estimate continuous variables. Values of P < 0.05 were considered to be statistically significant. The statistical analyses were performed using the Ekuseru-Toukei 2012^TM^ software program (Social Survey Research Information Co., Ltd., Tokyo, Japan).

### Data availability

The datasets generated during and analyzed during the current study are available from the corresponding author on reasonable request.

## Results

### The Patient characteristics and revascularization procedures

Table 1 shows the patients’ characteristics. The locations of the treated lesions were as follows: the iliac artery (n = 10), the common femoral artery (n = 1), the superficial femoral artery (n = 8), and a femoropopliteal bypass graft (n = 2). The lesions were occluded in four cases, the others were stenotic. The locations of the contralateral lesions were as follows: no obvious lesion (n = 11): iliac artery (n = 3): superficial femoral artery (n = 5): below the knee artery (n = 2). The presence of significant stenosis was defined >60% luminal narrowing. The lesions were occluded in two cases, the others were stenotic. The preoperative Rutherford category in the contralateral limbs were determined to be as follows: category 1 (n = 13), category 2 (n = 3), category3 (n = 4), category 5 (n = 1) (Table 1). The following procedures were performed: EVT (n = 17), thrombectomy and EVT (n = 2), femoropopliteal bypass procedure (n = 1), and thromboendarterectomy and EVT (n = 1). In the present study, EVT was performed through the ipsilateral femoral artery. Clinical success and technical success (residual stenosis of less than 30%) was achieved and the postoperative courses were uneventful in all cases. The perioperative treated limb ABI, TBI, and TP changed to a statistically significant extent (Table 2). The other hand, the perioperative contralateral ABI, TBI and TP did not change to any statistically significant extent (Table 2).

**Table 1:** Patient and treated limb characteristics.

	Subjects (n = 21)	
Median Age (years old) (IQR)	75 (71–77)	
Male/ Female	17/4	
Hypertension (%)	19 (90)	
Dyslipidemia (%)	13 (62)	
Coronary artery disease (%)	5 (24)	
Cerebrovascular disease (%)	4 (19)	
Diabetes (%)	9 (43)	
Chronic renal failure with hemodialysis (%)	3 (14)	
**Rutherford Category (treated limb)**	**Treated limb**	**Contralateral limb**
0–3	17	20
4	0	0
5	3	1
6	1	0

Abbreviations: IQR, interquartile range.

**Table 2:** Preoperative and postoperative ankle brachial pressure index, toe brachial pressure index, toe pressure.

	Preoperative (IQR^※^)	Postoperative (IQR)	P
**Treated limb**			
ABI	0.67 (0.44–0.75)	0.88 (0.85–1.01)	<0.001
TBI	0.46 (0.3–0.55)	0.6 (0.46–0.64)	0.005
TP	69 (40–85)	90 (77–99)	<0.001
**Contralateral limb**			
ABI	0.88 (0.69–0.95)	0.88 (0.80–0.95)	0.287
TBI	0.59 (0.44–0.66)	0.66 (0.46–0.74)	0.478
TP	87 (64–95)	103 (70–112)	0.687

Abbreviations: IQR, interquartile range; ABI, ankle brachial pressure index; TBI, toe brachial pressure index; TP, toe pressure.

### The evaluation of the bilateral feet skin perfusion using ICGA

**Figure 2:**
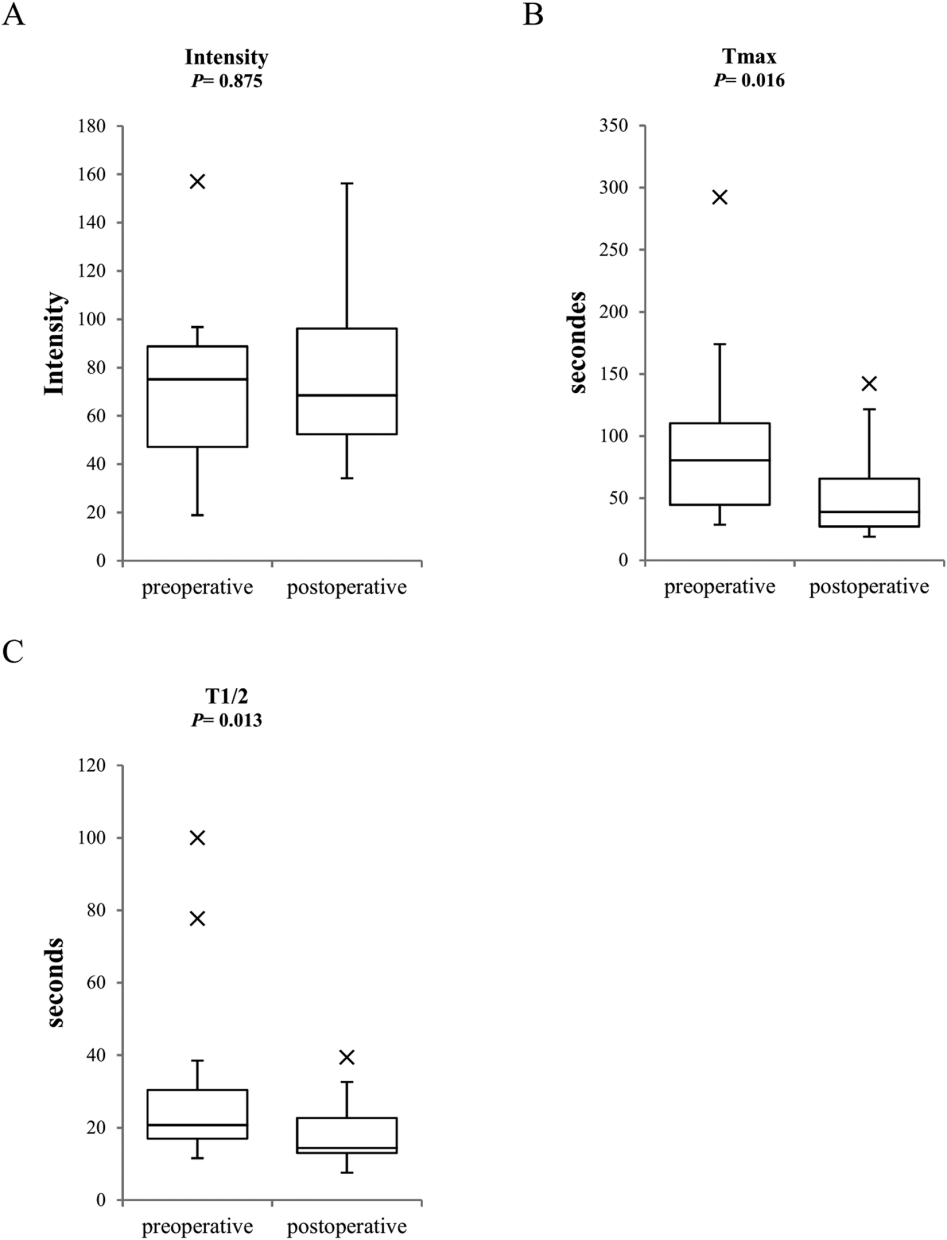
Preoperative and postoperative ICGA parameter values of the treated limb. The horizontal line in the middle of each box indicates the median; the top and bottom borders of the box mark the75th and 25th percentiles, respectively, the whiskers above and below the box extend 1.5 interquartile range in either direction, and the cross marks indicate outliers. *Note*. I_max_ indicated the magnitude of intensity from the onset of ICG to maximum intensity; T_max_ indicated the time from the onset of ICG to the maximum intensity, and the T_1/2_ indicated the time required to reach the half-maximum intensity from the onset.

**Figure 3:**
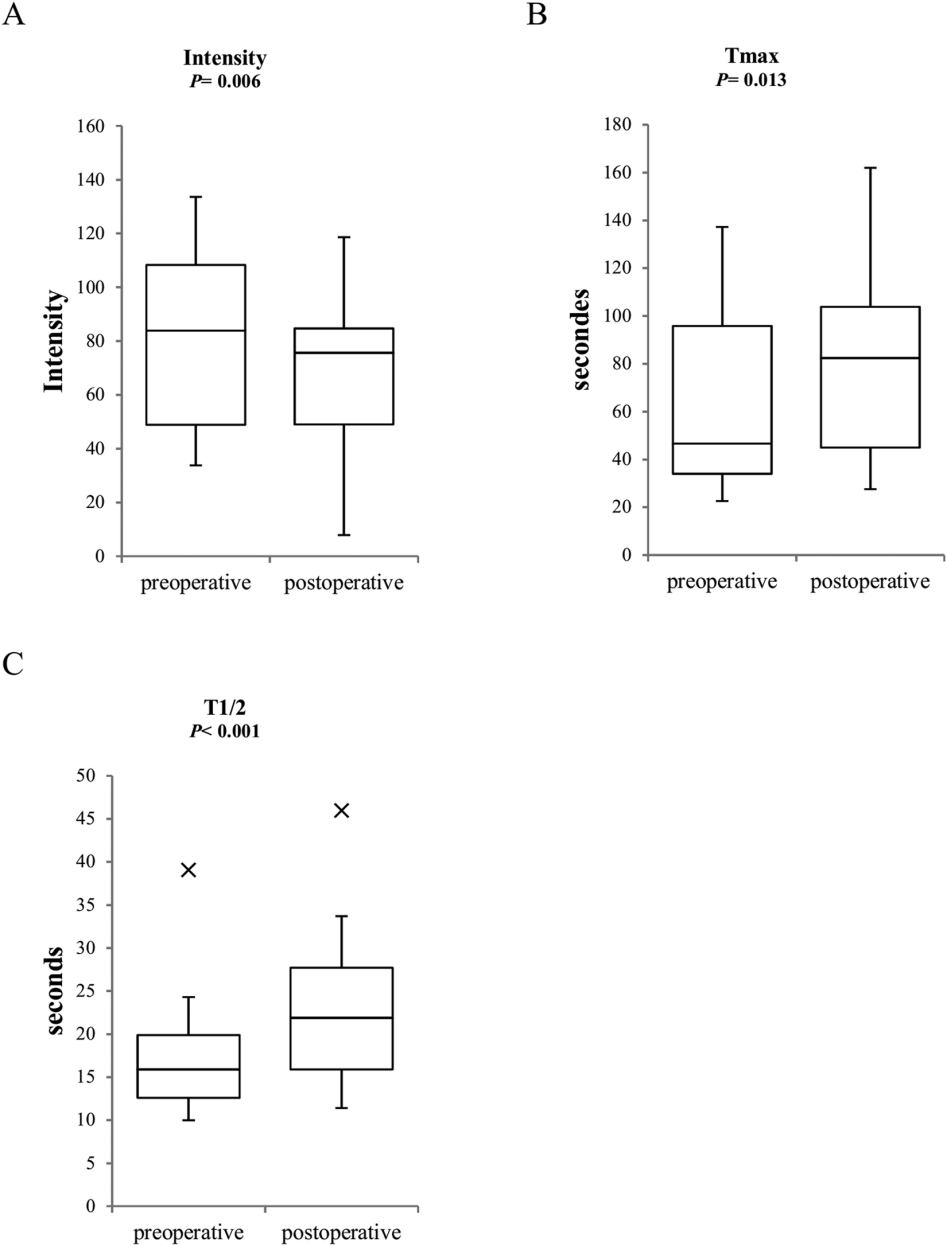
Preoperative and postoperative ICGA parameter values of the contralateral limb. The horizontal line in the middle of each box indicates the median; the top and bottom borders of the box mark the 75th and 25th percentiles, respectively, the whiskers above and below the box show the 1.5 interquartile range in either direction, and the cross marks indicate outliers. *Note*. I_max_ indicated the magnitude of intensity from the onset of ICG to maximum intensity; T_max_ indicated the time from the onset of ICG to the maximum intensity, and the T_1/2_ indicated the time required to reach the half-maximum intensity from the onset of ICG.

In the present study, the perfusion of bilateral feet were evaluated based on the results of preoperative and postoperative ICGA tests and the preoperative ABI exhibited a statistically significant correlation with the preoperative T_max_ and T _1/2_ (T_max_: ρ = −0.459, *P* = 0.003; T_1/2_: ρ = −0.401, *P* = 0.010). In the treated limb, the T_max_ and T_1/2_ values in the preoperative ICGA tests and postoperative were significantly different (Fig. 2A–C). In the contralateral limb, all the parameters of ICGA tests between preoperative and postoperative period were significantly different (Fig. 3A–C). Preoperative and postoperative T_1/2_ values in both limbs were more significantly different than T_max_ values. Therefore, we compared the RC of the contralateral T_1/2_ and the limb characteristics, including the RC of the ipsilateral T_1/2_, the ipsilateral ABI elevation and the preoperative contralateral limb. However, no statistically significant correlations were found (Table 3).

**Table 3:** Correlation between the rate of change in contralateral foot T_1/2_ and the limb characteristics.

	RC○ of Contralateral T_1/2_	ρ(Spearman)	P
RC of ipsilateral T_1/2_^×^		0.0273	0.906
Elevation in ipsilateral ABI^Δ^		−0.1880	0.379
Preoperative contralateral ABI		0.0718	0.738

Abbreviations: RC, rate of change; T_1/2_, the time required to reach the half-maximum intensity from the onset of ICG; ABI, ankle brachial pressure index.

## Discussion

Revascularization procedures are conducted to improve the ischemic symptoms associated with PAD. However, the effect of unilateral revascularization on the contralateral leg is unclear. The contralateral symptoms after the revascularization may be related with the progression of PAD. However, the previous reports suggested that the time needed for the progression of PAD is longer in untreated patients than in those who undergo unilateral revascularization. Nicoloff *et al*. reported that 22% of patients experienced the clinical progression of PAD (defined by a change in symptom or a need for surgical intervention) within a 5-year period^15^. Sigvant *et al*. also reported that 7% of asymptomatic PAD patients deteriorated intermittent claudication (IC) and that 21% of patients with IC progressed to CLI or IC deterioration in 5 years^16^. On the other hand, de Vries *et al*. reported that the cumulative percentage of patients with any contralateral symptoms (IC or CLI) ranged from 34.6% to 52.7% in the 4 years after the initial unilateral revascularization procedure^1^. These findings suggest that the progression of PAD is not the sole cause of the development of contralateral symptoms after unilateral revascularization.

In the present study, the ICGA tests showed that the perfusion of the contralateral foot significantly decreased and that there was no significant hemodynamic change after unilateral revascularization. Saucy *et al*. previously reported that laser Doppler imaging and plethysmography revealed similar results^2^. No patients experienced a deterioration of their contralateral leg symptoms in this short-term study period. However, Kang *et al*. reported that ICGA could be more effective for detecting mild functional impairment than ABI measurements^9^. Therefore, the decrease in the skin perfusion of the contralateral foot was probably related to the subsequent contralateral symptoms which occurred after unilateral revascularization. Because no factors were found to influence the effect of revascularization on the contralateral foot, it is difficult to predict the degree of the contralateral skin perfusion change. However, initial treatment for CLI and poor contralateral ABI have been reported to be associated with a high risk of contralateral CLI^1^ and ischemic condition observed in the contralateral limb should be carefully assessed when unilateral revascularization is performed.

Although the precise mechanisms underlying this phenomenon are unclear, we hypothesize that there are two mechanisms. The first possible mechanism is the steal phenomenon. In this study, the contralateral skin perfusion was observed in the early period after successful unilateral revascularization. In addition, it was observed in cases with no obvious PAD of the contralateral side. These facts suggest that revascularization stole blood from the contralateral leg. However, there might be a more complex or systemic mechanism because there was no significant correlation between the limb characteristics and the change in the perfusion in the skin of the contralateral foot. The second possible mechanism is related to the changes of some cytokines, such as nitric oxide (NO). An increased level of NO dilates the smooth muscle of the conduit arteries and maintains the concentration of NO at the luminal surface of the arterial endothelium, regardless of the flow increase^17^. However, Ural *et al*. reported that the serum level of NO measured at four weeks after revascularization was significantly lower than that in the preoperative period^18^. Additionally, Jacobi *et al*. suggested that endogenous NO increases the blood flow in the ischemic limb^19^.

The present study had some limitations. First, the study population was limited. However, we mainly reported that the ICGA tests showed that the perfusion of the contralateral foot significantly decreased. In that point, there were significant differences in the contralateral limb Tmax (P = 0.013), and T1/2 (P < 0.001) values on the perioperative ICGA tests. Second, we evaluated the contralateral foot perfusion shortly after revascularization surgeries, and the study period was short. Therefore it was unclear whether or not the change in the ICGA parameters was related to the contralateral symptoms and whether or not the decrease in the contralateral foot perfusion was a persistent phenomenon. These limitations merit further study.

In conclusion, our results indicate that unilateral revascularization decreases contralateral foot perfusion and the factors affecting this phenomenon are not clear. Although further studies are needed, we believe that a decrease in contralateral skin perfusion leads to the deterioration of the symptoms associated with PAD.
